# Cone-beam computed tomographic assessment of the inclination of the articular eminence in patients with temporomandibular disorders and chewing side preference

**DOI:** 10.1186/s12903-021-01760-4

**Published:** 2021-08-13

**Authors:** Junli Ma, Jiazhu Wang, Dongzong Huang, Zhaowu Wang, Min Hu, Hongchen Liu, Hua Jiang

**Affiliations:** 1 Department of Stomatology, General Hospital of Southern Theater of PLA, Guangzhou, People’s Republic of China; 2 Department of Stomatology, Chinese PLA General Hospital, No. 28 FuXing Road, Beijing, 100853 People’s Republic of China

**Keywords:** Cone-beam computed tomography, Chewing-side preference, Temporomandibular joint, Glenoid fossa, Articular eminence inclination, Temporomandibular disorders

## Abstract

**Background:**

Chewing side preference (CSP) has been proposed as one etiology of temporomandibular disorders (TMDs) as it can induce the structural changes of the temporomandibular joint. But its association with the inclination of the articular eminence (IAE) is unknown. This study aimed to compare IAE between patients with CSP and without CSP.

**Methods:**

Cone-beam computed tomography images of 90 patients with TMD (mean age of 45.6 years, 69 with CSP, 21 without CSP) and 20 participants without TMD and CSP (mean age of 41.3 years) were measured to compare IAE and depth of the glenoid fossa (DGF)

**Results:**

IAE and DGF showed a positive correlation among all the participants. Compared with the participants without TMD and CSP, the TMD patients without CSP presented a similar IAE but with a significantly higher value of DGF (*p* < 0.05); in contrast, the TMD patients with CSP presented a significantly greater IAE and DGF (*p* < 0.05). No bilateral differences in IAE and DGF were observed in all the participants. Except the male patients with CSP had a deeper fossa than did the female, no differences in IAE and DGF according to gender were observed.

**Conclusions:**

TMD patients with CSP seem to have a deep glenoid fossa with steep eminence which might be considered one characteristic imaging feature.

## Background

The glenoid fossa of the temporal bone is the upper part of the temporomandibular joint (TMJ), encompassing the joint disc and condyle with the attached joint capsules and ligaments. The posterior surface of the articular eminence bears the function load, and its inclination determines the path of condylar movement as well as the degree of rotation of the joint disc over the condyle during mouth movement [1].

Numerous studies have demonstrated that temporomandibular disorders (TMDs) are closely related to the morphologic changes of the TMJs [2–6]. As one indispensable part, the glenoid fossa undergoes persistent morphologic remodeling to better match with the joint disc and condyle due to the alteration of functional load, often associated with chewing habit, gender, food texture, and age [7].

Many studies have investigated the relationship between the morphology of the glenoid fossa, especially the inclination of the articular eminence (IAE), and TMDs. However, the results are contradictory. While some research revealed a steeper IAE in the TMD patients and attributed it to the development of TMDs [1, 8–10], other studies failed to support the relationship between the changes of IAE and TMDs [11–14].

Chewing side preference (CSP) or unilateral chewing, observed in 45–98% of the population [15], have been viewed as one potential contributor of TMDs [16–18]. Firstly, people with CSP have a higher prevalence of TMDs and also a great majority of TMD patients show CSP [16, 17, 19]. Secondly, the preferred chewing side often is the symptomatic side of the joints in TMD patients [20]. In addition, CSP could result into excessive load on the TMJ, leading to the anatomic and structural changes of TMJ, including cartilage [21], the glenoid fossa [22] and the condyle [23].

Great diversity in the methods and composition of participants may partially explain the controversies regarding IAE and TMDs. However, the presence or absence of CSP among the participants in previous studies was not mentioned. Considering the relationship between CSP and TMD, the remodeling of the TMJs during the development of TMDs, and the role of IAE played in the functional movement of the mandible, we speculate that CSP might be associated with the changes of IAE as one of the contributors of TMDs. Therefore, we enrolled TMD patients with CSP and without CSP to compare the imaging differences in the morphology of the fossa, aiming to explore the relationship between CSP and TMDs and provide another perspective to compare pervious imaging studies about IAE and TMDs.

## Methods

### Participants

This study was approved by the medical ethics committee of Chinese PLA Genera Hospital. A total of 90 patients who sought treatment of TMD in the department of stomatology at Chinese People’s Liberation Army General Hospital were randomly selected, and a written informed consent was obtained from each participant. The diagnose of TMD was made according to DC/TMD [19], the symptoms included unilateral joint pain, joint noise (clicking or crepitus) for more than 30 days. The exclusion criteria included masticatory muscle disorders, severe malocclusion, parafunction, traumatic injuries, congenital deformity, and a history of TMD treatment. Patients with systemic diseases that may affect the masticatory system, including diabetes, rheumatic disorders and gout, were also excluded.

The chewing preference was determined by self-report and a chewing gum test. A piece of chewing gum was placed on the center of the tongue dorsum; the direction towards which the gum was moved by the tongue in the first cycle of mastication was recorded as the preferred chewing side [23, 24]. Among 90 patients, 69 presented both TMD symptoms and CSP (the TC group), 21 presented only TMD symptoms (the T group). Other 20 asymptomatic volunteers without CSP were enrolled into the control group. All the participants were adult with a mean age of 44.8 years (Table 1).

**Table 1:** The composition by the age, gender and CSP of all the participants

	Age (years)		Gender		Total
			Female	Male	
Control	41.3 ± 13.1		12	8	20
T	46.5 ± 14.6		11	10	21
TC	45.3 ± 17.4	Left-CSP	23	9	32
		Right-CSP	23	14	37
Total	44.8 ± 15.6		69	41	110

### Imaging procedures and measurements

The image-acquiring and measurements were same as the procedures reported in our previous study [23]. CBCT scanning with a NewTom 3G flat panel-based Cone-beam computed tomography (CBCT) machine was performed, each participant was in a standard supine position with the mandible in a maximum intercuspal position and the Frankfort plane perpendicular to the horizontal plane. The scanning parameters were as follows, scanning time: 36 s with maximum output: 110 kV and 15 mAs, voxel size: 0.16 mm, typical exposure time: 5.4 s. FOV size:15 × 12 cm, voxel resolution: 0.25 mm, slices thickness: 0.5 mm. The central sagittal slice was chosen to measure IAE and the depth of the glenoid fossa (DGF).

**Fig. 1:**
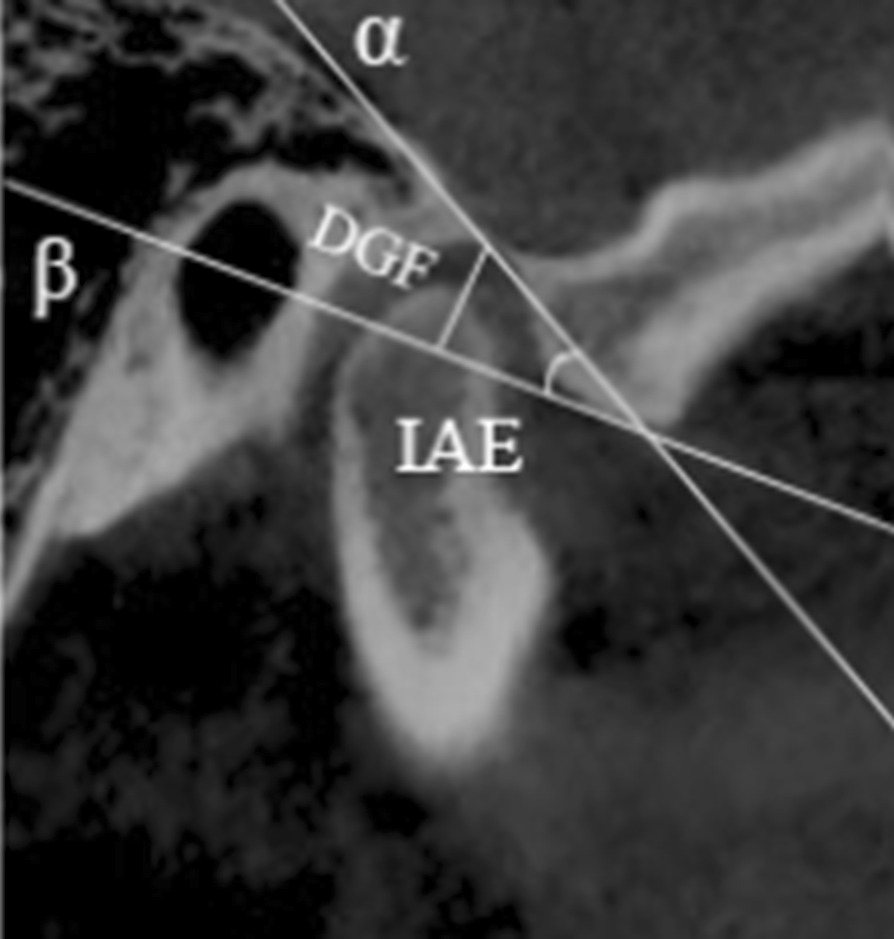
The inclination of articular eminence (IAE) and the depth of glenoid fossa (DGF). IAE: the angle between α and β line. DGF: the perpendicular distance between the highest point of the fossa and α-line

IAE was measured as the angle between two reference lines: the line through the highest point of glenoid fossa and the lowest point of articular eminence was set as α-line, and β-line was set as one through the midpoint of external ear foramen and the lowest point of articular eminence. DGF was the perpendicular distance from the highest point of joint fossa to β-line (Fig. 1).

### Statistical analysis

The distribution of genders and CSP were analyzed by Chi-squared test. The intergroup differences in IAE and DGF were compared with the analysis of variance (ANOVA). Intragroup differences in IAE and DGF according to side and gender were compared with paired *t*-test. Correlation analysis was performed to determine the relationship between IAE and DGF. All the analyses were performed using SPSS 16.0 (SPSS Inc., Chicago, IL, USA). The difference was considered significant when *p* ≤ 05 in all the analyses.

## Results

### The demographic characteristics of participants

Table 1 shows the demographic characteristics and the presence of CSP of all the participants. Twenty asymptomatic participants without CSP (8 males and 12 females) were included in the control group with a mean age of 41.3 years. The T group comprised 21 TMD patients without CSP (mean age of 46.5 years), and the TC group consisted of 69 TMD patients with CSP (mean age of 45.3 years), 32 with left CSP and 37 with right CSP. The Chi-squared test showed a nonsignificant difference in the gender distribution between 3 groups (*x*^2^ = 1.415, *p* = 0.176); in the TC group, the distribution of left CSP and right CSP according to genders also showed no significant differences (*x*^2^ = 0.728, *p* = 0.276).

The symptomatic side was in accordance with the preferred chewing side in 69.6% of patients (48/69) in the TC group. There was a significant concordance between the preferred chewing side and the symptomatic side (the Pearson *x*^2^ = 9.59, *p* = 0.002; *k* = 0.37; *p* = 0.002).

### The intergroup comparison of IAE and DGF

**Fig. 2:**
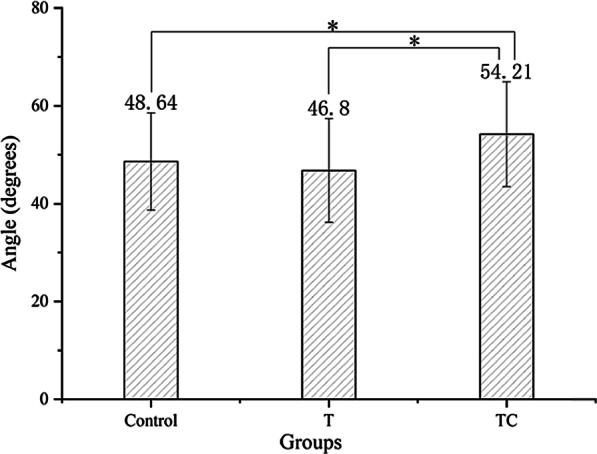
The comparisons of IAE between groups

ANOVA results showed a significant difference in IAE between 3 groups (*p* < 0.05). The patients with CSP had a significantly greater value of IAE than those in the T and control groups (*p* < 0.05). But there was no significant difference between the T and control groups (*p* > 0.05; Fig. 2).

**Fig. 3:**
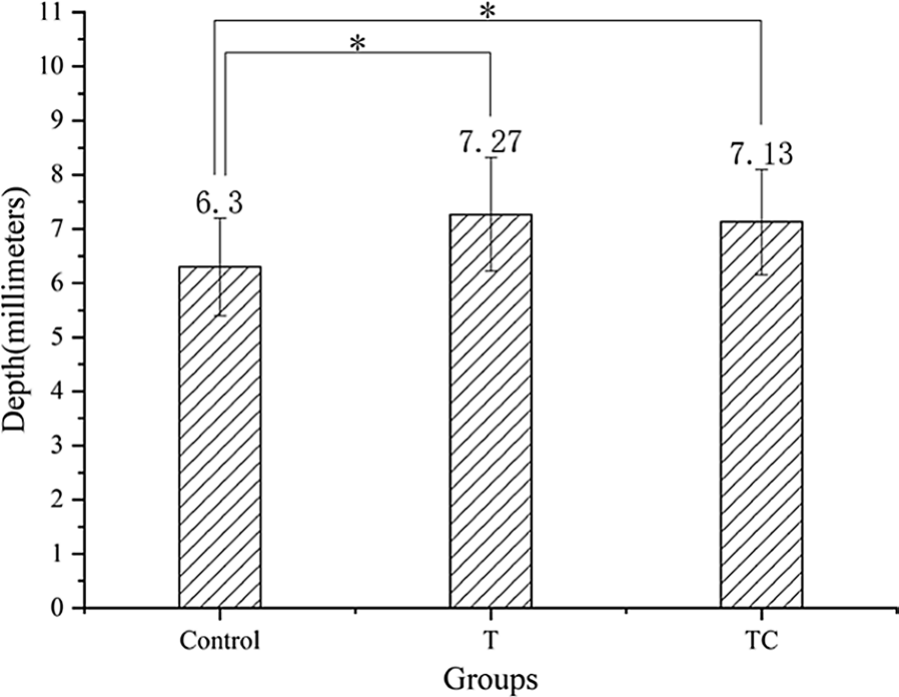
The comparisons of DGF between groups

The DGF also exhibited a significant difference between groups. The TMD patients in the TC and T group had a deeper fossa compared with that in the asymptomatic participants (*p* < 0.05). No significant difference between the T and TC groups was observed (*p* > 0.05; Fig. 3).

**Fig. 4:**
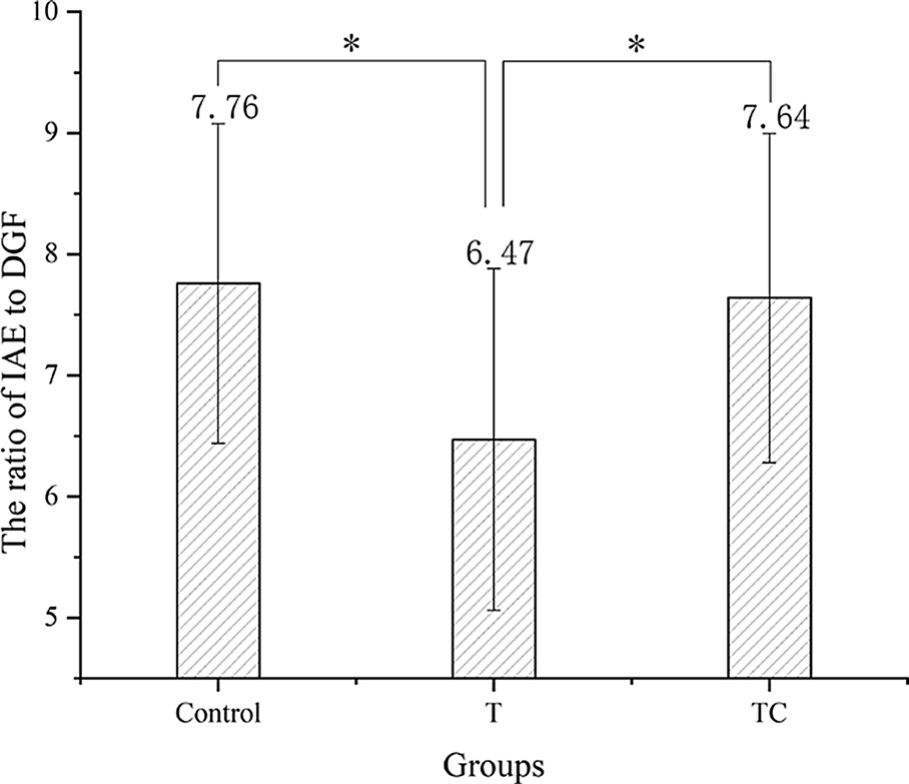
The ratio of IAE to DGF among each group

Figure 4 shows the ratio of IAE/DGF in the all the participants. The participants in the TC group and the control group exhibited similar ratio without significant difference (*p* > 0.05), but the ratio in the T group was significantly lower than that in other groups (*p* < 0.05).

### Intragroup analysis

Neither IAE nor DGF showed significant differences bilaterally within the control and T groups (*p* > 0.05). The preferred side of joints in the TC group also presented similar values of IAE and DGF as the unpreferred side (*p* > 0.05; Table 2).

**Table 2:** Bilateral comparisons of IAE and DGF within each group

	IAE (°)		*p*-value	DGF (mm)	*p*-value
Control	Left	47.95 ± 11.07	0.234	6.32 ± 0.92	0.881
	Right	49.34 ± 8.90		6.29 ± 0.90	
T	Left	46.09 ± 10.94	0.447	7.40 ± 1.11	0.099
	Right	47.51 ± 10.48		7.14 ± 1.00	
TC	P-side	53.90 ± 10.68	0.666	7.13 ± 1.00	0.988
	U-side	54,51 ± 10.79		7.13 ± 0.95	

The comparison of IAE between genders within each group showed no significant differences (*p* > 0.05); except that the male TMD patients with CSP presented significantly greater DGF than the female counterparts (*p* < 0.05), no differences in DGF between genders were observed within other groups (*p* > 0.05; Table 3).

**Table 3:** The differences in IAE and DGF between genders within each group

		Male	Female	*p*-value
Control	IAE	49.22 ± 8.07	48.25 ± 11.16	0.768
	DGF	6.52 ± 0.97	6.15 ± 0.84	0.214
T	IAE	48.93 ± 9.87	44.87 ± 11.12	0.220
	DGF	7.51 ± 1.10	7.06 ± 0.98	0.619
TC	IAE	56.10 ± 10.93	53.26 ± 10.52	0.143
	DGF	7.52 ± 1.13	6.93 ± 0.82	0.001

### Correlation analysis

With all the participants included or within each group, a strong positive correlation was observed between IAE and DGF (*p* < 0.05). The highest *r* value of 0.559 was observed in the control group, and the TC presented the second highest *r* value of 0.483 (Table 4).

**Table 4:** The pearson correlation between IAE and DGF

	IAE	DGF	*r-*value	*p*-value
Control	48.64 ± 9.93	6.3 ± 0.90	0.559	0.000
T	46.80 ± 10.61	7.27 ± 1.05	0.457	0.002
TC	54.21 ± 10.70	7.13 ± 0.97	0.483	0.000
All	51.78 ± 10.98	7.01 ± 1.03	0.472	0.000

## Discussion

The articular eminence is exposed to functional load during chewing, and its shape is in a constant remodeling to match the movement of the condyle and joint disc. Therefore, the articular inclination reflecting the condylar path might also be influenced by the changing jaw movement and functional load. In our previous work, we observed the eminence in the preferred side was steeper than that in the unpreferred side among the healthy subjects with CSP. But the presentation of IAE in TMD patients with CSP is unknown [23]. In the present study, we found a steep eminence and deep fossa in the TMD patients with CSP. In contrast, the TMD patients without CSP showed a deep fossa without steep IAE. This result indicates that CSP has an influence on IAE among patients with TMD. In addition, no bilateral differences in IAE were observed in all the participants. To our best knowledge, similar results have not been reported.

Santana et al. [25] observed that TMD patients with CSP presented a steeper condylar path angle in the symptomatic side of joints compared with that in the contralateral side. They considered CSP as one etiology of TMD and proposed a new term, ‘‘habitual chewing side syndrome’’, to replace the nonspecific symptom-based ‘‘temporomandibular joint disorders’’. Although condylar path does not equal to IAE, but they are positively correlated with each other [26]. Therefore, our findings provided imaging evidence supporting the phenomenon observed by Santana et al., and this deep fossa with steep eminence might be viewed as one imaging feature for TMD patients with CSP. We also observed that IAE was positively correlated with DGF, which is in line with previous reports [1, 9].

In the present study, we did not find TMD patients without CSP had a steeper eminence compared with the subjects in the control group. This is in agreement with the results of Ren et al. [11], Pullinger et al. [12] and Imanimoghaddam et al. [14], but contradictory to Al-Rawi et al. [1], Sato et al. [8] and Paknahad et al. [10]. These studies showed a steeper eminence in the TMD patients than in the heathy individuals. In contrast, Sümbüllü et al. [13] found IAE in TMD patients was lower than it was in the healthy subjects.

Although many factors might be related to the controversies above, including gender, TMD severity, type of TMDs, imaging modality and measuring methods, no definitive conclusion could be made. For instance, Al-Rawi et al. [1] reported that only the male TMD patients had a greater IAE, female patients had no such a presentation. But other studies failed to observe similar result [10, 13, 27, 28]. In our study, the TC group included more female patients, which might cause bias. But the comparison between genders within each group showed no differences in IAE. Thus, gender might not be a strong factor influencing IAE.

Age might also be a factor influencing the results. The adults have a greater IAE compared with children [28]. However, the articular eminence is relatively stable without significant changes due to aging after it achieves complete development by the age of 20 to 30 years [29]. In our study, all the enrolled participants were adults and showed similar age distribution between groups, eliminating the possible bias caused by age.

Variations in TMD type have been related to different changes of TMJ morphology. Sülün et al. [9] and Rabelo et al. [30] reported that only patients with disc displacement with reduction (DDWR) presented greater IAE, which was not observed in the event of disc displacement without reduction. However, Poluha et al. [31] failed to observe the difference in IAE between the normal joints and joints with DDWR in their MRI study. Ren et al. [11] found a greater IAE in the normal joints rather than the joints with disc displacement, thus, they did not support a steep eminence as one predisposing factor for disc displacement. In addition, some studies provided no clear information about the types of TMDs, causing more difficulties in comparing the various results [1, 12–14].

Measuring methods could directly influence the results. Two reference lines must be defined to measure IAE in imaging study. One is the line depicting the posterior surface of eminence, most studies adopted “best-fit line” [1, 9, 11, 12, 31] or “top-roof line” [10, 30, 32]. The other one is usually the line parallel to Frankfort plane, but other lines have also been reported [8, 10, 12]. Different reference lines directly affect the value of IAE and the final results. Sato et al. [8] reported a greater IAE in joints with DDWR when measuring was taken with the “best-fit line” method, but with the “top-roof line” method, no difference was observed.

Regarding DGF, we found TMD patients with or without CSP presented a greater value than did the control. Only the male patients with CSP showed a deeper fossa than the female counterparts, other participants showed no difference in DGF between genders. Rabelo et al. [30] compared the DGF between TMD patients with different types of dis displacement and found no differences in a MRI study, but box-shaped eminences presented greater DGF. Paknahad et al. observed that only the male TMD patients had a deeper DGF than female, the normal population had no such a difference between genders [10]. These findings indicate that impacts of gender on IAE or DGF are conditional and related to other factors, including the presence of TMDs and chewing habit.

The articular eminence forms the anterior limits for the movement of condyle and disc, thus, a deep glenoid fossa with a steep eminence means that both the condyle and disc have to move forward and downward further than the movement in a flat and shallow fossa during mouth opening. Therefore, the disc has a greater chance of being captured during mouth movement, predisposing individuals to disc displacement [1, 26, 30]. In this regard, our findings support previous research that showed a positive relation between CSP and internal derangement of TMJ [33–35].

As for why the TMD patients with CSP had such a deep fossa and greater IAE, we speculate that the change of condyle position and increased load are the direct factors. Because the working side condyle is in a posterior and superior position during normal chewing movement [36, 37]; the individuals with CSP would constantly repeat this position in the preferred side of joints rather than alternating with the balancing side, causing excessive load and the following adaptive changes of the joints [36, 38]. The osseous remodeling secondary to disc displacement might also be another possibility. However, only at an advanced stage-disc displacement without reduction, could obvious osseous changes present [39–41], which might partially explain why the patients without CSP showed similar IAE as healthy individuals if most of our patients were with disc displacement with reduction.

The limitation of the present study is we could not give a definitive assertion whether a steep eminence with a deep fossa is the reason or merely a consequence of TMDs by the imaging results. Considering multiple factors associated with TMDs, we could not rule out the possibility that differences in the morphology of the glenoid fossa between groups were also related to other factors besides CSP, like parafunction, types and severity of TMD, or occlusion. Although it is difficult to control multiple factors in a clinical study with a large sample size, more homogeneous participants and uniform measuring method should be taken into the consideration when designing the study to evaluate the relationship between a specific factor and TMDs. Within the limits of the present study, a deep glenoid fossa with steep eminence might be taken as one characteristic imaging presentation for the TMD patients with CSP. CSP is a predisposing factor for the development of TMDs.

## Conclusions

Within the limits of the present study, a deep glenoid fossa with steep eminence might be taken as one characteristic imaging presentation for the TMD patients with CSP. CSP is a predisposing factor for the development of TMDs.

## Data Availability

The datasets used and/or analysed during the current study are available from the corresponding author on reasonable request.

## Abbreviations

CSP: Chewing side preference
IAE: Inclination of the articular eminence
TMDs: Temporomandibular disorders
DGF: Depth of the glenoid fossa
TMJ: Temporomandibular joint
CBCT: Cone-beam computed tomography
ANOVA: Analysis of variance
DDWR: Disc displacement with reduction
